# Aspirin for preeclampsia prevention in low- and middle-income countries: mind the gaps

**DOI:** 10.1016/j.xagr.2024.100352

**Published:** 2024-04-03

**Authors:** Ellen Kupka, James M. Roberts, Zaleha A. Mahdy, Carlos Escudero, Lina Bergman, Leandro De Oliveira, Global Pregnancy Collaboration

**Affiliations:** a Department of Obstetrics and Gynecology, Institute of Clinical Sciences, Sahlgrenska Academy, University of Gothenburg (Drs Kupka and Bergman), Sweden; b Department of Research and Higher Education, Center for Clinical Research Dalarna, Uppsala University, Region Dalarna (Dr Kupka), Falun, Sweden; c Magee-Womens Research Institute (Dr Roberts), Pittsburgh, PA; d Department of Obstetrics and Gynecology, Epidemiology and Clinical and Translational Research, University of Pittsburgh (Dr Roberts), Pittsburgh, PA; e Department of Obstetrics and Gynaecology, University Kebangsaan Malaysia Medical Center (Dr Mahdy), Cheras, Malaysia; f Vascular Physiology Laboratory, Basic Sciences Department, Faculty of Sciences, Universidad del Bio-Bio (Dr Escudero), Chillan, Chile; g Group of Research and Innovation in Vascular Health (GRIVAS Health) (Dr Escudero), Chillan, Chile; h Department of Women's and Children's Health, Uppsala University (Dr Bergman), Uppsala, Sweden; i Department of Obstetrics and Gynecology, Stellenbosch University (Dr Bergman), Cape Town, South Africa; j São Paulo State University (UNESP), Medical School (Dr Oliveira), Botucatu

**Keywords:** aspirin dosage, aspirin in preeclampsia, low- and middle-income countries, low-dose aspirin, low-resource settings, maternal and perinatal mortality, preeclampsia prevention, preeclampsia screening

## Abstract

Preeclampsia is a syndrome that continues to be a major contributor to maternal and neonatal mortality, especially in low-income countries. Low-dose aspirin reduces the risk of preeclampsia, but the mechanism is still unknown. Risk factors to identify women at risk of preeclampsia are based on clinical characteristics. Women identified as high-risk would benefit from aspirin treatment initiated, preferably at the end of the first trimester. Current efforts have largely focused on developing screening algorithms that incorporate clinical risk factors, maternal biomarkers, and uterine artery Doppler evaluated in the first trimester. However, most studies on preeclampsia are conducted in high-income settings, raising uncertainties about whether the information gained can be totally applied in low-resource settings. In low- and middle-income countries, lack of adequate antenatal care and late commencement of antenatal care visits pose significant challenges for both screening for preeclampsia and initiating aspirin treatment. Furthermore, the preventive effect of first-trimester screening based on algorithms and subsequent aspirin treatment is primarily seen for preterm preeclampsia, and reviews indicate minimal or no impact on reducing the risk of term preeclampsia. The lack of evidence regarding the effectiveness of aspirin in preventing term preeclampsia is a crucial concern, as 75% of women will develop this subtype of the syndrome. Regarding adverse outcomes, low-dose aspirin has been linked to a possible higher risk of postpartum hemorrhage, a condition as deadly as preeclampsia in many low- and middle-income countries. The increased risk of postpartum hemorrhage among women in low-income settings should be taken into consideration when discussing which pregnant women would benefit from the use of aspirin and the ideal aspirin dosage for preventing preeclampsia. In addition, women's adherence to aspirin during pregnancy is crucial for determining its effectiveness and complications, an aspect often overlooked in trials. In this review, we analyze the knowledge gaps that must be addressed to safely increase low-dose aspirin use in low- and middle-income countries, and we propose directions for future research.

## Introduction

Preeclampsia is an obstetric complication that affects 3% to 8% of pregnant women. Together with hemorrhage, they are the leading cause of mortality for mothers and infants in low-resource settings.^1^^,^^2^ Every year, up to 70,000 women worldwide lose their lives, and 500,000 pregnancies result in stillbirths or neonatal deaths because of preeclampsia.^3^ Hemorrhage is responsible for another 70,000 maternal deaths each year.^1^ This is accompanied by an even greater excess of morbidity for affected women.

Meta-analyses have reported that the use of low-dose aspirin reduces the risk of preeclampsia by 18% to 62%.^4^^,^^5^ In addition, low-dose aspirin has also been associated with a reduction in stillbirths and neonatal deaths.^5^ Although some studies have found an association between low-dose aspirin and a decreased risk of a small-for-gestational-age infant,^5^ others have not found any such association.^4^^,^^6^^,^^7^ The latest Cochrane systematic review from 2019 showed that low-dose aspirin should ideally be initiated before 20 weeks of gestation for a protective effect against preeclampsia.^5^ Another review has suggested that even initiation before 16 weeks of gestation is preferable.^8^ This is a challenge in some low and middle-income countries (LMIC) in which women might have limited access to antenatal care.^9^ One of the most significant risk reductions for preterm preeclampsia was reported from the Aspirin for Evidence-based Preeclampsia Prevention (ASPRE) trial.^8^ This study was conducted in high-income settings, using a combined screening algorithm that included clinical risk factors, maternal biomarkers, and uterine artery Doppler. The intervention used 150 mg of aspirin, and quite importantly, the risk reduction was restricted to the 25% of women with preeclampsia who delivered preterm (<37 weeks). However, a secondary analysis of the ASPRE trial suggested that aspirin, in fact, delays the disease onset from preterm to term.^10^ Term preeclampsia is not benign and exerts long-lasting consequences on both mother and their children, in particular in LMIC.^4^ The interest in prescribing higher doses of aspirin is increasing.^11^^,^^12^ At the same time, a growing body of evidence suggests an association between low-dose aspirin use and postpartum hemorrhage.^5^ Higher aspirin dosages might increase the risk of bleeding complications, including neonatal hemorrhage. However, in low-resource settings, where hemorrhage poses the highest risk of morbidity and mortality, reliable information is extremely limited.

**Figure 1:**
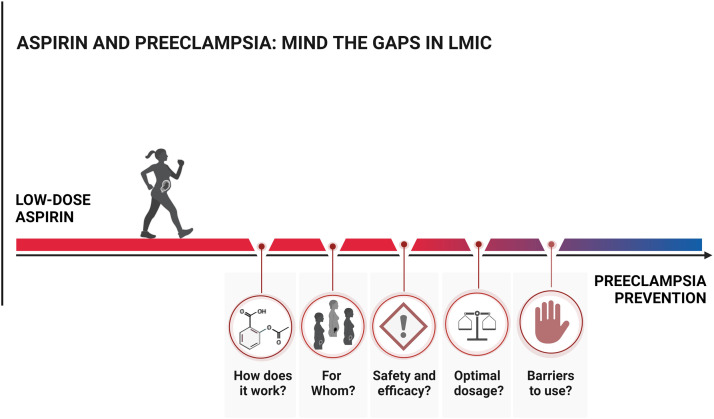
Aspirin and preeclampsia: mind the gaps LMIC There are knowledge gaps concerning the use of low-dose aspirin in LMIC. They include aspirin's mechanism, how to identify women who benefit from aspirin, safety and efficacy, optimal aspirin dosage, and adherence to guidelines and treatment. Addressing and bridging these gaps is essential to safely enhance aspirin therapy in LMIC. *LMIC*, low and middle-income countries.

It is common to assume that information gained in studies in high-resource settings can be applied to low-resource settings. This might not be applicable regarding aspirin usage for preeclampsia. In this review, we analyze the existing gaps in knowledge concerning the use of low-dose aspirin in LMIC, including mechanisms of action, screening methods, safety, optimal dosage, adherence, and effectiveness. Addressing these gaps is essential to safely enhance aspirin therapy in LMIC and ultimately reduce maternal and perinatal mortality (Figure 1).

*Kupka.* Aspirin and preeclampsia: mind the gaps*. Am J Obstet Gynecol Glob Rep 2024.*

## How does aspirin reduce preeclampsia?

Aspirin has been proposed as a therapeutic intervention for preeclampsia since the early 1980s.^13^ The initial target effect was to reduce thromboxane without reducing prostacyclin. This would provide an antithrombotic effect. Nevertheless, even with the positive effects of low-dose aspirin therapy in restoring the placental tromboxane or prostacyclin balance,^14^ many women treated with aspirin will still develop preeclampsia. Other possible mechanisms include improvement of placentation^15^ and a reduction of endothelial cell dysfunction.^16^ However, the underlying course of action for aspirin-prevention of preeclampsia remains unclear. Therefore, it is not possible, other than empirically by outcome, to guide aspirin dosage in the setting of preeclampsia prevention.

## Who should take aspirin?

Various screening methods for early pregnancy have been proposed to predict the maternal risk of developing preeclampsia and identify women who would benefit from aspirin use. Screening based on clinical risk factors is the most common approach. However, screening based on the National Institute for Health and Care Excellence (NICE) guideline has been reported to detect only 30% of all cases of preeclampsia and 41% of preterm preeclampsia cases.^17^ This has led to great debate on the implementation of multivariable prediction models that add maternal biomarkers and uterine artery Doppler to the traditional clinical risk factors.^18^

It has been demonstrated that combined screening algorithms demonstrate better test performance for preeclampsia requiring early delivery, typically before 37 weeks of gestation, compared with preeclampsia with later onset or all preeclampsia.^18^ However, as disease prevalence decreases, the positive predictive value declines. For rare conditions like preterm preeclampsia, the positive predictive value is notably low.^19^ International guidelines vary in their recommendations for screening methods. When accessible, the first-trimester combined test^20^ is recommended by the International Society for the Study of Hypertension and The International Federation of Gynecology and Obstetrics (FIGO) for universal screening.^11^^,^^12^ The latter acknowledges the challenges of implementing such a strategy in low- and middle-income countries but refers to prospective studies that have validated the effectiveness and predictive performance of the test, primarily in high-income countries (HIC).^19^^,^^21^, ^22^, ^23^, ^24^ In contrast, the American College of Obstetricians and Gynecologists (ACOG) and the NICE highlight limited evidence for the accuracy of predictive models, advocating for clinical risk factor-based screening (Table).^12^^,^^18^^,^^25^, ^26^, ^27^, ^28^, ^29^, ^30^, ^31^, ^32^, ^33^ A few guidelines on preeclampsia prevention are available from middle-income countries,^34^ and they are usually based on international recommendations (Table^12^^,^^18^^,^^27^, ^28^, ^29^, ^30^, ^31^, ^32^, ^33^). There is a lack of accessible guidelines from LMIC that are adapted to their local settings and population.

**Table:** Preeclampsia guidelines

	ACOG^27^	NICE^28^	ISSHP^29^	FIGO^12^	SOMANZ^a^^.^^30^	FOGSI^31^	SASOG^32^	RBEHG^33^
Clinical risk factors								
Chronic hypertension	High	High	High	Included	High	3 points	High	High
Type 1 or type 2 diabetes	High	High	High	Included	High	3 points	High	High
Renal disease	High	High	High	Included	High	3 points	High	High
Autoimmune disease (SLE, APLS)	High	High	High	Included	High	3 points	High	High
History of preeclampsia	High	High	High	Included	High	2 points	High	High
Multifetal gestation	High	Moderate	Moderate	Included	High	2 points	Moderate	High
History of other pregnancy hypertensive disorder	Not included	High	Not included	Included	High	2 points	High	Not included
Use of assisted reproductive technology	Moderate	Not included	High	Included	Moderate	Oocyte donation or surrogacy: 3 points IVF/ICSI: 1 point	High	Moderate
Body mass index	>30 kg/m^2^: moderate	≥35 kg/m^2^: moderate	>30 kg/m^2^: moderate	Included	≥35 kg/m^2^: moderate	>30 kg/m^2^: 1 point; >35 kg/m^2^: 2 points Excessive weight gain during pregnancy: 1 point	≥35 kg/m^2^: moderate	>30 kg/m^2^: high
Blood pressure	Not included	Not included	Not included	Not included	SBP >130 mmHg and/or DBP >80 mmHg: moderate	Median artery pressure >85: 1 point	Not included	Not included
Nulliparity	Moderate	Moderate	Moderate	Included	Moderate	1 point	Moderate	Moderate
Family history	Of preeclampsia (mother or sister): moderate	Of preeclampsia (mother or sister): moderate	Not included	Of preeclampsia (mother or sister): included	Of preeclampsia (mother or sister): moderate	Of cardiovascular disease: 1 point Woman born as SGA: 1 point	Of preeclampsia (mother or sister): moderate	Of preeclampsia (mother or sister): moderate
Pregnancy interval	>10 y: moderate	>10 y: moderate	Not included	>10 y: included	>10 y: moderate	>7 y: 1 point	>10 y: moderate	>10 y: moderate
Short duration of sperm exposure	Not included	Not included	Not included	Not included	Not included	1 point	Not included	Not included
Maternal age (age)	>35 y: moderate	≥40 y: moderate	≥40 y: moderate	Included	≥40 y: moderate	>35 y or <19 y: 1 point	≥40 y: moderate	>35 y: moderate
Maternal height	Not included	Not included	Not included	Included	Not included	Not included	Included	Not included
Obstetric history (LBW, SGA, or previous adverse pregnancy outcome)	Moderate	Not included	Moderate	Included	Not included	Not included	Included	Moderate
Other maternal conditions	Not included	Not included	Not included	Not included	Not included	Mental disorder: 3 points Thrombophilia: 3 points Gestational diabetes mellitus: 2 points Hypothyroidism: 2 points Anemia: 1 point Polycystic ovary syndrome: 1 point Chronic vascular disease (dyslipidemia): 1 point	Not included	Not included
Low socioeconomic status	Moderate	Not included	Not included	Included	Not included	Not included	Included	Moderate
Black race (as a proxy for underlying racism)	Moderate	Not included	Not included	Included	Not included	Not included	Included	Moderate
Recommendations for aspirin prophylaxis								
When to offer aspirin	The presence of any high-risk factor or the presence of any 2 moderate-risk factors	The presence of any high-risk factor or the presence of any 2 moderate-risk factors	Presence of any high-risk factor or presence of any 2 moderate-risk factors or high-risk on the Fetal Medicine Foundation first-trimester combined test	High-risk on the Fetal Medicine Foundation first-trimester combined test	The presence of any high-risk factor or the presence of any 2 moderate-risk factors	A total score of ≥3	High-risk on the Fetal Medicine Foundation first-trimester combined test, or if not possible to use, presence of any high-risk factor or presence of any 2 moderate-risk factors	The presence of any high-risk factor or the presence of any 2 moderate-risk factors
Universal first trimester screening with multivariable prediction model	Does not recommend universal first-trimester screening	Does not recommend universal first-trimester screening	Supports universal first-trimester screening	Supports universal first-trimester screening	Conditionally supports the first trimester screening when accessible	Does not recommend universal first-trimester screening	Supports universal first-trimester screening	Does not recommend universal first-trimester screening
Recommended daily dose of aspirin	81 mg initiated between 12 and 28 wk of gestation, ideally before 16 wk	75–150 mg from 12 wk of gestation	Women screened with Fetal Medicine Foundation first-trimester combined test: 150 mg before 12 wk of gestation Women screened with risk factors: 100–162 mg before 16 wk of gestation	150 mg initiated between 11 and 14 wk of (+6 days) gestation	150 mg before 16 wk of gestation	75–150 mg before 12 wk of gestation	Women screened with Fetal Medicine Foundation first-trimester combined test: 150 mg before 12 wk of gestation Women screened with risk factors: 75 mg before 12 wk of gestation	100 mg from 12 wk of gestation
When to cease aspirin	Continue until delivery	Continue until delivery	Continue until 36 wk of gestation	Continue until 36 wk of gestation, delivery, or when preeclampsia is diagnosed	Cessation of aspirin between 34 wk of gestation and delivery	Continue until 2 days before delivery	75 mg: until delivery 150 mg: until 36 wk of gestation	Continue until 36 wk of gestation

Adapted from Chappell et al,^18^ 2021.

*ACOG*, American College of Obstetricians and Gynecologists; *APLS*, antiphospholipid syndrome; *ASOG*, The South African Society of Obstetricians and Gynaecologists; *FIGO*, The International Federation of Gynecology and Obstetrics; *FOGSI*, Federation of Obstetric and Gynecological Societies of India; *ICSI*, Intracytoplasmic sperm injection; *ISSHP*, International Society for the Study of Hypertension in Pregnancy; *IVF*, in vitro fertilization; *LBW*, low birthweight; *NICE*, National Institute for Health and Care Excellence; *RBEHG*, Brazilian Network For The Study Of Hypertension In Pregnancy; *SGA*, small-for-gestational-age; *SLE*, systemic lupus erythematosus; *SOMANZ*, Society of Obstetric Medicine of Australia and New Zealand.

a Guideline still underrevision.

The use of multivariable prediction algorithms is a challenge in LMIC. There are important limitations regarding infrastructure, costs, and access to necessary equipment at antenatal healthcare facilities. FIGO recommends that in LMIC settings, prediction algorithms incorporating at least maternal characteristics, medical history, and blood pressure measurements should be employed. This can increase the detection rate compared with risk factor-based screening. However, many women fail to attend antenatal care in early pregnancy.^9^^,^^35^ Ultrasonographic assessment of gestational age was available in 38% of facilities, but in some countries, availability was as low as <10%. These factors pose a challenge to screening and initiating treatment with low-dose aspirin early in pregnancy in LMIC. In addition, it is unknown if risk factors for preeclampsia in HIC align with those in LMIC, considering potential unique factors such as poor healthcare access and high rates of diseases such as malaria and human immunodeficiency virus.^36^^,^^37^ For instance, there is an association between malaria infections in early pregnancy and defective placentation, potentially leading to hypertensive disorders, intrauterine growth restriction, and preterm birth.^38^ A recent substudy of the ASPIRIN trial, a randomized multicountry study in 6 LMICs, found that women with malaria in early pregnancy experienced higher rates of perinatal mortality than women without malaria when administered low-dose aspirin.^39^ Hence, the applicability of risk models developed in HIC to LMICs needs to be assessed carefully.

There has been a suggestion to implement universal aspirin prescription during pregnancy. However, there is no evidence to endorse such a recommendation. A clinical decision analysis study from 2019 evaluated the cost-effectiveness of various models of preeclampsia screening and aspirin prophylaxis strategies.^23^ The model assumed 100% adherence and that aspirin use was only associated with 2 possible side effects: gastrointestinal bleeding and aspirin-exacerbated respiratory disease. Universal aspirin administration was linked to a reduction in preeclampsia cases and lower overall costs than in scenarios in which no aspirin was administered or in which aspirin was given based on biomarkers and ultrasound measures or only clinical risk factors.^23^ However, it is unlikely that 100% compliance would be maintained with universal aspirin prescription. As a response, another analysis was performed under the assumption of greater compliance among women at high risk of preeclampsia.^40^ In this analysis, selectively prescribing aspirin was more efficient and also more cost-effective. These different results raise the controversial aspects of the universal use of low-dose aspirin. Importantly, none of these analyses considered the risk of postpartum hemorrhage.

However, one of the greatest difficulties in many LMICs is limited access to antenatal care. Without access to antenatal care in the first trimester, neither universal aspirin prescription nor universal screening based on complex algorithms is feasible. Improving access to antenatal care is an immense challenge but would have many benefits apart from making first-trimester screening and aspirin initiation possible.

## Is aspirin safe in pregnancy?

For decades, the use of low-dose aspirin in pregnancy has been considered safe.^41^ However, previous research on low-dose aspirin and bleeding events has been scarce and controversial. Bleeding events, and in particular postpartum hemorrhage, have been difficult to study in meta-analyses because of uncertainties in the assessment of blood loss, bleeding often being an exploratory endpoint, different definitions of postpartum hemorrhage, and different national or organizational guidelines for the approach to postpartum hemorrhage. Importantly, some studies have suggested that low-dose aspirin use is associated with an increased risk of bleeding complications during pregnancy and, in particular, postpartum.^5^^,^^42^^,^^43^ However, there is no randomized controlled trial that has investigated bleeding events as a primary outcome after low-dose aspirin use in pregnancy.

The 2019 Cochrane Systematic Review of antiplatelet agents for the prevention of preeclampsia included 77 trials and 40,249 women using aspirin at a dosage of 50–150 mg.^5^ Three large trials included women from upper-middle-income countries, whereas no large trials were conducted in low-income countries. The occurrence of postpartum hemorrhage >500 mL was slightly increased (23,769 women, 19 trials; relative relative risk [RR], 1.06 [95% CI, 1.00–1.12]). Nonetheless, the quality of evidence for this outcome was downgraded to moderate because of differences in measurements of blood loss. In addition, the authors suggested that antiplatelet agents probably marginally increase placental abruption.^5^

Following the 2019 Cochrane meta-analysis, a more recent meta-analysis from 2023 was published that focused on postpartum hemorrhage in women using low-dose aspirin. The authors reported an increased risk of postpartum hemorrhage among women using low-dose aspirin (odds ratio [OR], 1.20 [95%, CI, 1.07–1.34]).^42^ It contained 21 studies, including 17 randomized controlled trials. The increased risk remained when only randomized controlled trials were retained (OR, 1.12 [95% CI, 1.00–1.25]), but the estimate was unsure, ranging between 0% to 25% increased risk.^42^ Five studies originated from LMIC, 14 studies were from HIC, and 3 studies involved multiple-country collaboration. The average dosage of aspirin was 81 mg, with a range from 60 to 150 mg, and the definition of postpartum hemorrhage and time for cessation of aspirin differed across studies. The association between aspirin use and postpartum hemorrhage was stronger in HIC. The cause for this is unknown but might be attributed to reduced adherence to aspirin and challenges in measuring blood loss in LMIC.

A systematic review from 2014, including 27,032 neonates to women using 60–150 mg aspirin, found no association between low-dose aspirin use in pregnancy and neonatal intracranial hemorrhage. In most of these trials, aspirin treatment was continued until delivery, and the predominant aspirin dosages were 60 or 100 mg.^44^ The Cochrane Systematic Review reported that low-dose aspirin use was associated with no risk of intraventricular hemorrhage (RR, 0.99 [95% CI, 0.72–1.36]) and neonatal bleeding (RR, 0.90 [95% CI, 0.75–1.08]) among 26,184 neonates in 10 trials.^5^

Hence, robust data suggest a higher risk of postpartum hemorrhage with aspirin use during pregnancy, whereas the link with neonatal intracranial hemorrhage is unclear. Low-dose aspirin use has been associated with an increased risk of placental abruption and intrapartum bleeding.^5^^,^^45^ However, there are conflicting data, with some studies reporting no association.^46^

Most of these studies were conducted in HIC, and it remains uncertain whether these results apply to low-resource settings. The ASPIRIN trial reported no difference in postpartum hemorrhage >1000 mL between women using aspirin 81 mg (0.9%) and placebo (0.7%). However, the study population consisted of healthy nulliparous women, and the reported incidence of postpartum hemorrhage was unexpectedly low, questioning the accuracy in reporting bleeding outcomes.^6^ It is possible that the resulting morbidity and mortality would be worse in LMIC without rapid access to emergency obstetric care. Complications like anemia further increase the risk of postpartum hemorrhage and death in LMICs.^47^ In HIC, maternal deaths because of hemorrhage and preeclampsia are comparable; however, in LMIC, this is not always true. Although hemorrhage and hypertension equally contribute to maternal mortality in Latin America and the Caribbean, hemorrhage accounts for more than twice as many direct maternal deaths as preeclampsia in Asia and Northern Africa.^1^ However, it is important to note that many women who died because of hemorrhage also had preeclampsia.

Thus, there is an urgent need for large, randomized trials in LMIC that investigate bleeding complications after aspirin use in pregnancy as a primary outcome alongside the protective effects against preeclampsia and its related maternal complications to determine whether the benefits outweigh the harms in these settings.

## What is the optimal aspirin dosage?

The question about the optimal aspirin dosage for preeclampsia prevention is still unresolved. International guidelines recommend 75–150 mg per day and vary between countries and regions (Table).^12^^,^^18^^,^^25^, ^26^, ^27^, ^28^, ^29^, ^30^, ^31^, ^32^, ^33^^,^^48^

In the 2019 Cochrane Systematic Review, 80% of women received aspirin 50–75 mg, and 20% received 100–150 mg.^5^ Although not statistically significant, there was an indication that a higher dosage (≥75 mg) may be more effective than doses <75 mg. In trials using individual participant data, the RR of preeclampsia was 0.92 for women using aspirin <75 mg, although the CI included the possibility of no effect (22,618 women, 11 trials; RR, 0.92 [95% CI, 0.85–1.00]). For women using aspirin ≥75 mg, the RR of preeclampsia was 0.78 (9107 women, 16 trials; 95% CI, 0.66–0.92) in trials using individual participant data.^5^ A systematic review and meta-analysis from 2017 reported a dose-response effect for the prevention of preeclampsia when aspirin treatment (50–150 mg) was initiated at ≤16 weeks. Higher dosages of aspirin were associated with greater risk reduction. However, out of 21 included studies, only one study from 1985 used 150 mg of aspirin.^49^ A 2018 study analyzing individual participant data found decreasing rates of preterm preeclampsia with increasing dosage of aspirin but with overlapping confidence intervals.^50^ In this study, the use of 100 mg aspirin resulted in the lowest predicted rate of preterm preeclampsia of 1.6%.^50^ Nevertheless, a recent meta-analysis favored 150–162 mg over 75–80 mg for preventing preterm preeclampsia,^51^ although limited by few studies and methodological issues.^52^ If a higher dose proves to be more effective, the impact would be greater for women in LMIC than in HIC. Preventing preeclampsia and eclampsia is even more urgent in LMICs, where antenatal care is not always available, access to emergency obstetric services might be limited, and magnesium sulfate is often unavailable or underused.^53^ In addition, survival rates for preterm infants are significantly lower in LMIC than in HIC.^54^

The benefit of preeclampsia prevention with increasing dosage must be weighed against the potential bleeding risk. Although higher doses might be more effective, there is concern about whether higher doses increase bleeding complications. This is a very important consideration in LMIC, in which aspirin-associated hemorrhage is likely to be more consequential. In a worst-case scenario, low-dose aspirin would increase maternal mortality because of increased bleeding risk in low-income settings.

Trials assessing the association between low-dose aspirin use and bleeding complications have mainly utilized aspirin dosages below 100 mg,^5^ and it is essential to confirm the safety of doses exceeding 100 mg in low-resource settings. The ASPRE study did not include postpartum hemorrhage as a primary or secondary outcome, limiting data for assessing bleeding risks with higher dosages (150 mg).^4^

To resolve the question about the optimal aspirin dosage for preeclampsia prevention, large trials comparing dosages in LMIC assessing both efficacy in preventing preeclampsia and risk of bleeding complications are necessary.

## Is there adherence to current guidelines regarding screening and treatment?

To prevent preeclampsia in the clinical setting, 2 conditions must be met. First, the obstetric care provider must adhere to the guidelines for preeclampsia prevention and advise women at risk to use low-dose aspirin. Second, the woman needs to be compliant with the therapy.

Although low-dose aspirin to prevent preeclampsia has been indicated mainly on the basis of the presence of high-risk conditions, the prescription of low-dose aspirin has been described as underused. Retrospective cohort studies from the United States and England, in which risk factors-based screening was employed, found that only 30% of women identified as high-risk for preeclampsia received recommendations for aspirin use.^48^^,^^55^

In addition, women's adherence to aspirin treatment during pregnancy is essential for defining both its effectiveness and potential complications. Despite this importance, most trials have not addressed this topic appropriately, and there is a gap in research regarding aspirin adherence among pregnant women in LMIC. A 2017 review of randomized trials from HIC and LMIC in cardiology and obstetrics found that only 37% (25 out of 68) of obstetric trials and 32% (20 out of 62) of cardiology trials referred to aspirin adherence. It is important to keep in mind that poor adherence not only compromises the success of trials but also can mask adverse effects. The reported aspirin adherence differs between studies. In the ASPRE trial, 80% of participants exhibited an adherence rate of ≥85%, correlating with a decreased risk of preterm preeclampsia.^4^ However, medical adherence is likely greater in a randomized controlled trial compared with real-world settings. A 2020 Australian prospective cohort study found that only 56% of high-risk women were adherent, defined as having a higher than 90% adherence rate.^56^ Adherent women experienced lower rates of preeclampsia, intrauterine growth restriction, and preterm birth. A prospective multicenter study in England reported that women screened with the NICE guidelines risk factor-based screening had a compliance rate of only 23%.^17^ Postpartum hemorrhage was not assessed in either study.

In the nonobstetric literature, adherence to chronic medication is generally lower in LMIC than in HIC.^57^ Numerous factors serve as barriers to medication adherence in LMICs. They include reliance on traditional medicine, religious beliefs, limited communication with healthcare providers, financial constraints, limited healthcare access, and challenges related to medication availability.^58^ Interventions such as mobile health services and facility-based interventions have the potential to increase adherence to antenatal healthcare.^59^ However, this intervention may be difficult to implement in low-resource settings. To increase the utilization of low-dose aspirin among women at risk of preeclampsia, it is crucial to assess the perspectives and beliefs of both pregnant women and obstetric healthcare providers in these settings.

## Concluding remarks and perspective

Reducing the incidence of preeclampsia is a crucial step toward decreasing maternal and neonatal mortality in LMICs. However, this cannot come at the expense of an increased risk of bleeding complications. The implementation of aspirin prophylaxis in LMIC is challenging because of economic constraints, lack of necessary equipment, and impairments in providing or attending to antenatal care. The ideal aspirin dosage for preeclampsia prevention remains uncertain. Although higher doses of aspirin might be more effective, there are some concerns that they could raise the likelihood of bleeding events. Another question concerns the timing of aspirin use during pregnancy. Although there is a prevailing belief that it must be started before the 16th week of pregnancy, not all meta-analyses support this view.^60^

**Figure 2:**
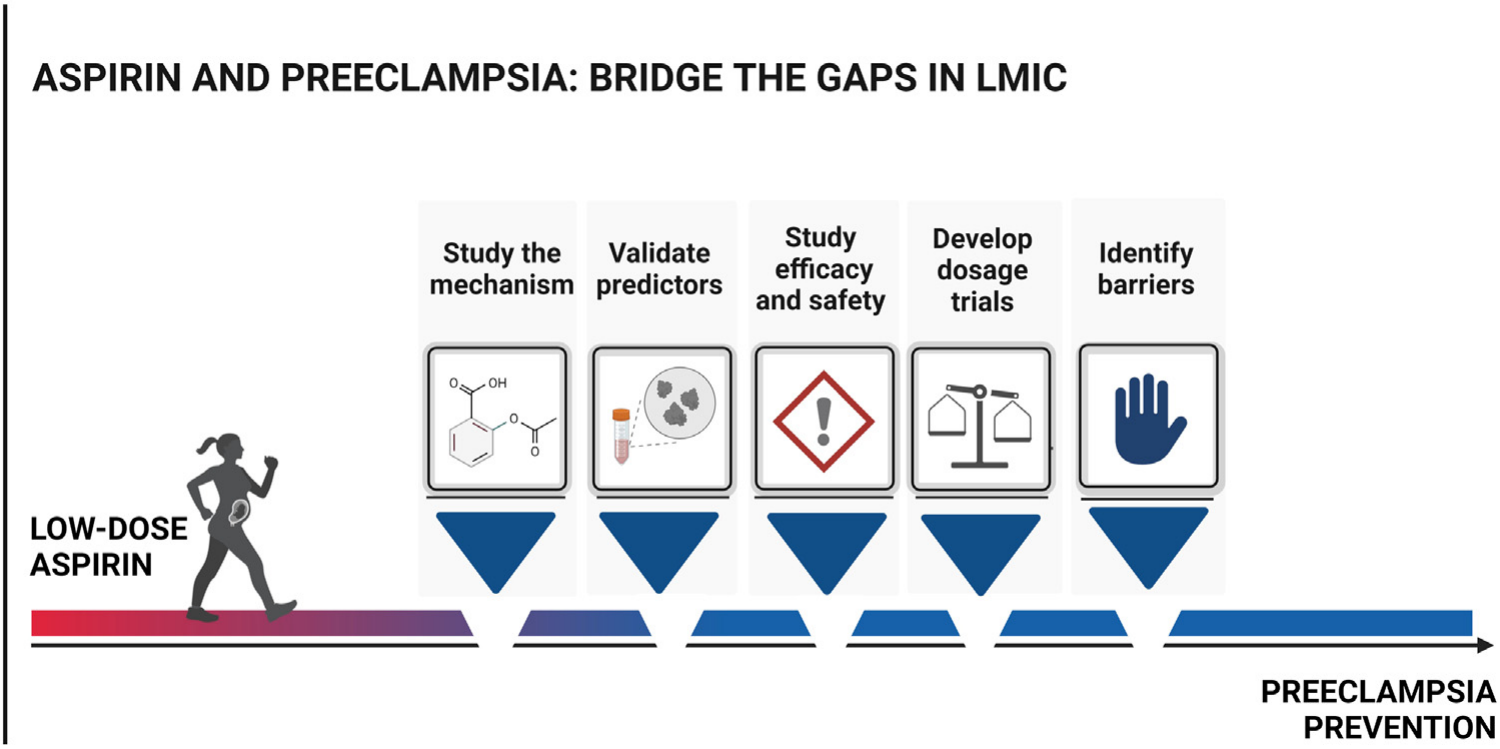
Aspirin and preeclampsia: bridge the gaps in LMIC To bridge the knowledge gaps concerning the use of low-dose aspirin in LMIC, we suggest further studies on aspirin's mechanism of action, predictors for screening in LMIC, safety and efficacy, the effects of different dosages of aspirin in randomized trials, and barriers to aspirin use. *LMIC*, low and middle-income countries.

In addition, the adherence of both healthcare providers and pregnant women to aspirin therapy is essential. To safely increase aspirin therapy in LMIC and reduce the burden of preeclampsia, we recommend the investigation of the following research areas for appropriate decisions (Figure 2):

• Incorporate research on aspirin's mechanism of action in clinical trials.
• Identify and validate predictors that are pertinent and feasible in LMIC contexts to use as screening tools.
• Study aspirin's efficacy and safety as primary outcomes in LMIC.
• Trial different dosages of aspirin and measure bleeding as a main outcome in aspirin trials in LMIC.
• Identify barriers and facilitators to antenatal care in LMIC and work with remote solutions to increase adherence and the number of visits in antenatal care.
• Evaluate women's views regarding compliance and what outcomes matter to women with a specific focus on LMIC.

*Kupka.* Aspirin and preeclampsia: mind the gaps*. Am J Obstet Gynecol Glob Rep 2024.*

The importance of building research capacity in LMICs has long been recognized. However, research challenges in LMICs persist. There is often limited access to scientific infrastructure, including adequate access to clinical data and scientifically trained personnel, and funding constraints.^61^ The use of mobile phone applications has shown potential to facilitate data collection in LMIC.^53^ These applications could serve as valuable tools for self-reporting pregnancy outcomes and complications, such as bleeding during aspirin therapy, and for collecting data on predictors of preeclampsia. In addition, they can play a significant role in educating women about the importance of antenatal care, improving communication with healthcare providers, and collecting data on barriers and facilitators to attending antenatal care.

## CRediT authorship contribution statement

**Ellen Kupka:** Writing – review & editing, Writing – original draft. **James M. Roberts:** Writing – review & editing, Writing – original draft. **Zaleha A. Mahdy:** Writing – review & editing, Writing – original draft. **Carlos Escudero:** Writing – review & editing, Writing – original draft. **Lina Bergman:** Writing – review & editing, Writing – original draft. **Leandro De Oliveira:** Writing – review & editing, Writing – original draft.
